# Pediatric HIV^+^ Kaposi sarcoma exhibits clinical, virological, and molecular features different from the adult disease

**DOI:** 10.1172/jci.insight.167854

**Published:** 2023-11-22

**Authors:** Carolina Caro-Vegas, Alice Peng, Angelica Juarez, Allison Silverstein, William Kamiyango, Jimmy Villiera, Casey L. McAtee, Rizine Mzikamanda, Tamiwe Tomoka, Erin C. Peckham-Gregory, Razia Moorad, Carrie L. Kovarik, Liane R. Campbell, Parth S. Mehta, Peter N. Kazembe, Carl E. Allen, Michael E. Scheurer, Nmazuo W. Ozuah, Dirk P. Dittmer, Nader Kim El-Mallawany

**Affiliations:** 1 UNC Lineberger Comprehensive Cancer Center and Center for AIDS Research, Chapel Hill, North Carolina, USA.; 2 Texas Children’s Cancer & Hematology Center Global HOPE (Hematology-Oncology Pediatric Excellence) Program Malawi, Lilongwe, Malawi.; 3 University of Colorado, Department of Pediatrics, Denver, Colorado, USA.; 4 Baylor College of Medicine (BCM), Department of Pediatrics, Houston, Texas, USA.; 5 Texas Children’s Hospital Cancer & Hematology Center, Houston, Texas, USA.; 6 University of North Carolina Project-Malawi, Kamuzu Central Hospital Pathology Laboratory, Lilongwe, Malawi.; 7 University of Pennsylvania, Philadelphia, Pennsylvania, USA.; 8 BCM International Pediatric AIDS Initiative Children’s Foundation Tanzania, Mbeya, Tanzania.; 9 BCM International Pediatric AIDS Initiative Children’s Foundation Malawi, Lilongwe, Malawi.

**Keywords:** AIDS/HIV, Oncology, Cancer, Kaposi sarcoma

## Abstract

**BACKGROUND:**

Kaposi sarcoma (KS) is among the most common childhood cancers in Eastern and Central Africa. Pediatric KS has a distinctive clinical presentation compared with adult KS, which includes a tendency for primary lymph node involvement, a considerable proportion of patients lacking cutaneous lesions, and a potential for fulminant disease. The molecular mechanisms or correlates for these disease features are unknown.

**METHODS:**

This was a cross-sectional study. All cases were confirmed by IHC for KS-associated herpesvirus (KSHV) LANA protein. Baseline blood samples were profiled for HIV and KSHV genome copy numbers by qPCR and secreted cytokines by ELISA. Biopsies were characterized for viral and human transcription, and KSHV genomes were determined when possible.

**RESULTS:**

Seventy participants with pediatric KS were enrolled between June 2013 and August 2019 in Malawi and compared with adult patients with KS. They exhibited high KSHV genome copy numbers and IL-6/IL-10 levels. Four biopsies (16%) had a viral transcription pattern consistent with lytic viral replication.

**CONCLUSION:**

The unique features of pediatric KS may contribute to the specific clinical manifestations and may direct future treatment options.

**FUNDING:**

US National Institutes of Health U54-CA-254569, PO1-CA019014, U54-CA254564, RO1-CA23958.

## Introduction

Childhood cancers are infrequent in the US and Europe, with an incidence of 13–16 cases per 100,000 children overall and leukemia being the most common diagnosis. The situation is starkly different in sub-Saharan Africa (SSA), where Kaposi sarcoma-associated herpesvirus (KSHV) is endemic. KSHV is transmitted from mother to child before puberty. In SSA, early-age immune challenges, such as malaria and, more recently, HIV infection, are common. Burkitt lymphoma is the most recognized pediatric viral cancer in SSA, ever since Denis Burkitt described it in 1958 in Uganda and Michael Anthony Epstein and Yvonne Barr associated this endemic childhood neoplasia with Epstein-Barr virus (EBV) (reviewed in ref. 1).

Kaposi sarcoma (KS) emerged as an AIDS-defining disease in the US and Europe in the 1980s (2). It was associated with KSHV in 1994 (3). KSHV, or human herpesvirus 8 (HHV-8), is the etiologic agent of all types of KS. This includes HIV^–^ classic KS and pediatric KS (HIV^+^ and HIV^–^) (4). KSHV was endemic in SSA before the emergence of HIV. In SSA, seroconversion peaks before puberty, and a connection with malaria exposure is suspected (5, 6). Since HIV began to spread in SSA, KS has become the most common cancer in males living with HIV in Malawi, Uganda, and other KSHV-endemic countries.

Pediatric KS is one of SSA’s most common childhood cancers, with a median incidence of 67.35 per 100,000 HIV-infected children (7–9), as 90% of the world’s 2 million children living with HIV are in SSA. Many acquired HIV through mother-to-child transmission before the introduction of antiretroviral therapy (ART) to prevent mother-to-child transmission (10). Mother-to-child transmission of KSHV seems less common (11). ART-treated HIV-infected children acquire KSHV infection at the same rate and mount similar serological responses as their HIV-uninfected peers (12). Today, there is better ART coverage in general, including with pregnant women who are living with HIV and who are started on ART to prevent transmission. This decreased the number of children infected with HIV through vertical transmission. Nonetheless, KS still occurs in children and adolescents living with HIV, those who are on ART, and those who are not on ART (13). The effect of the 3-year COVID-induced disruption of HIV care on pediatric KS rates is unclear.

Pediatric KS can have a similar clinical presentation as adult KS, with visceral, oral, and skin lesions (14); however, pediatric KS has additional characteristics (15–17). Many cases present with primary lymph node involvement, some with lymphadenopathy alone, while others present with symptoms seen in KSHV inflammatory cytokine syndrome (KICS) (18). Severe cytopenia is frequent in patients with lymphadenopathic or visceral disease. Currently, there are no universal treatment guidelines for pediatric KS. Treatment recommendations are extrapolated based on adult KS or small retrospective studies (19–22). Our limited knowledge about the molecular pathobiology of pediatric KS (HIV^+^ and HIV) constitutes a gap in our knowledge that this study aims to close.

Earlier, we proposed a pediatric-specific KS classification (23). Pediatric KS is divided into 4 groups: cutaneous/oral, lymphadenopathic, woody edema, and visceral and/or disseminated cutaneous/oral. It is unknown if these groups represent distinct stages of the disease or are different clinical representations of the same underlying biology. We also observed laboratory correlates of KICS in some children, such as increased levels of KSHV viral load, IL-6, and IL-10 (24–27). Overall, diagnostic options are limited, as most pediatric patients live in low to middle-class countries (LMICs) with a lack of infrastructure and resource constraints. Therefore, studies focusing on pediatric KS are vital to improving the prognosis for these patients.

This report describes a cohort of 70 pediatric patients with KS enrolled between June 2013 and August 2019 at Kamuzu Central Hospital in Lilongwe, Malawi. Clinical and laboratory conditions are compared with adult KS encountered at the same location and time. This comparison highlights the broad spectrum of pediatric KS clinical presentation, typically higher KSHV viral load but a similar transcription profile. We also obtained the first 11 complete KSHV genomes from pediatric KS tumors and showed they cluster with adult KSHV strains from the region. These studies provide biological plausibility to transferring adult treatment regimens, such as pomalidomide and paclitaxel (28, 29), to pediatric KS but point to the need to monitor KSHV and cytokine levels.

## Results

### Clinical presentation.

**Figure 1:**
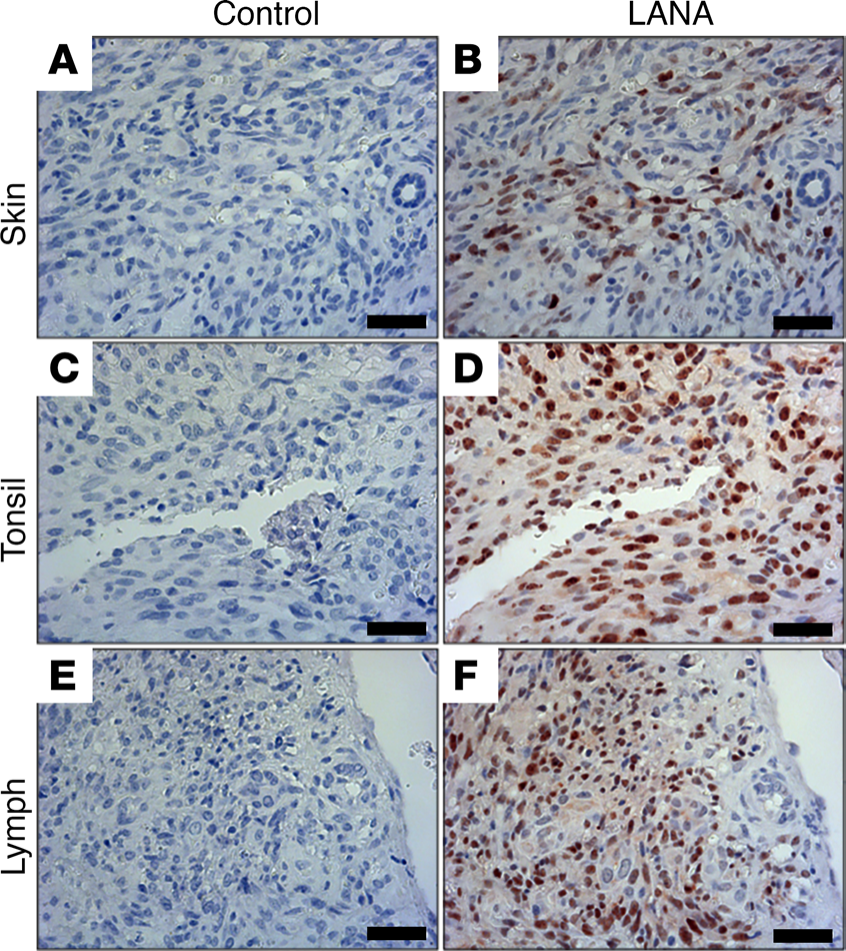
LANA staining of pediatric KS biopsies. Biopsies from different locations, (**A** and **B**) skin, (**C** and **D**) tonsil, and (**E** and **F**) lymph node, were LANA stained to confirm KS diagnosis. Results for all cases with available blocks for confirmation are presented in Supplemental Figure 1. Scale bar: 50 μm; original magnification at ×20.

Seventy participants with pediatric KS were enrolled between June 2013 and August 2019 at Kamuzu Central Hospital in Malawi. Diagnostic biopsies were subjected to LANA staining to confirm the KS diagnosis. All samples, regardless of the biopsy site, skin, lymph, or tonsil, showed robust LANA staining (Figure 1 and Supplemental Figure 1; supplemental material available online with this article; https://doi.org/10.1172/jci.insight.167854DS1). HIV status was ascertained per Malawi guidelines using a rapid test or PCR.

The characteristics of the cohort are presented in Table 1. The median age was 6.7 years, and 42% were male. Table 1 also shows the distribution of KS presentations classified as per our earlier schema (23). Class 1A is defined as only having a mild cutaneous or oral KS, while class 1B is a moderate case; 1 (1.4%) and 8 (11.4%) participants fell into these groups, respectively. Class 2 is determined by lymph node involvement. This class represents most cases with 37 participants (52.9%); class 3 is defined as having woody edema, which was seen in 11 (15.7%) cases. Visceral lesion and/or disseminated cutaneous or oral KS was present in 13 (18.6%) cases (Table 1). A total of 50 (71.4%) patients had lymph node involvement, while only 29 (41.4%) had skin lesions. Supplemental Tables 1 and 2 show the clinical characteristics of HIV^–^ (*n* = 14) and HIV^+^ (*n* = 56) participants separately.

### Pediatric KS has a different viral presentation than adult KS.

**Figure 2:**
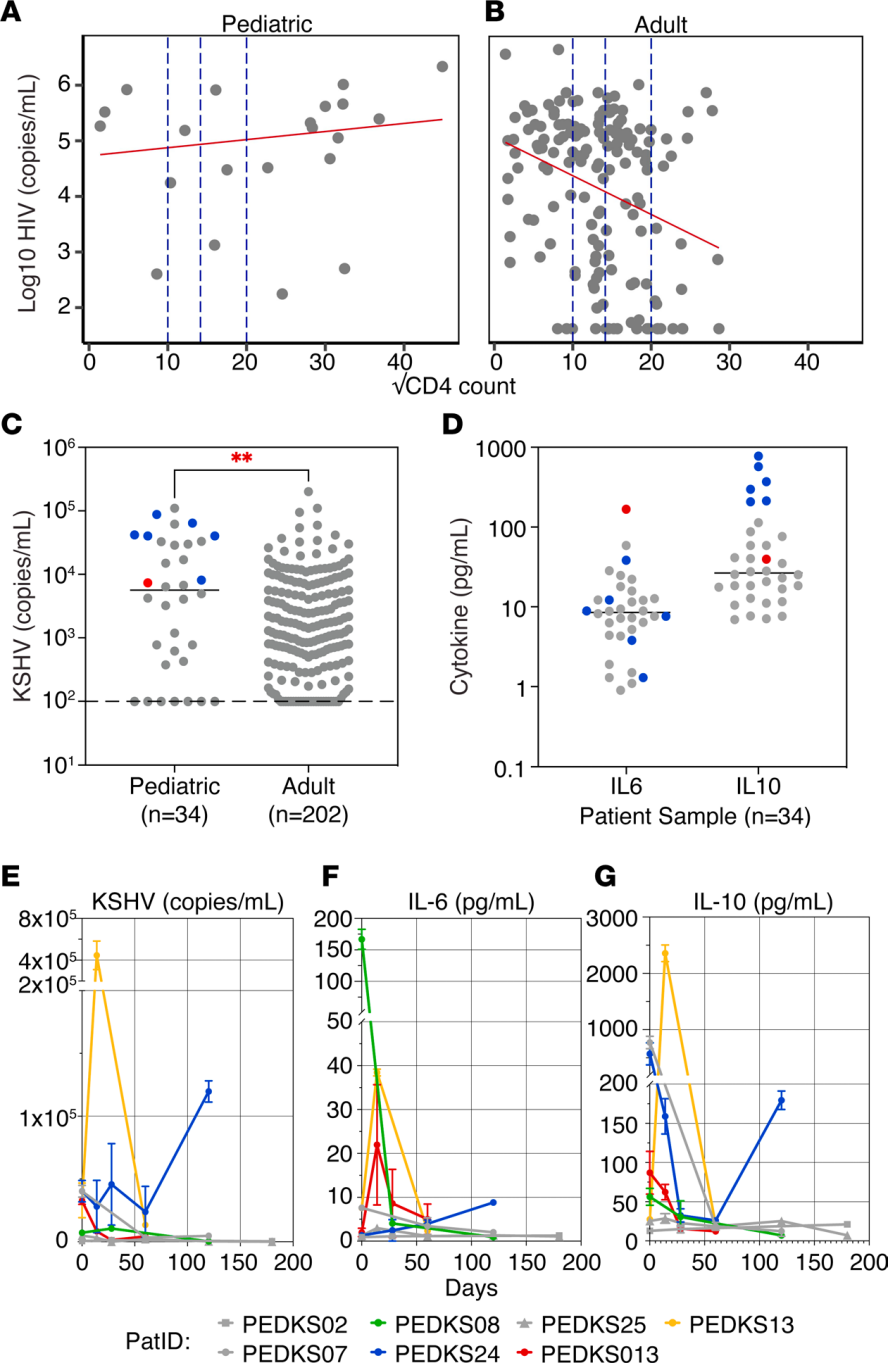
Systemic viral and cytokine levels in pediatric KS. HIV viral load versus CD4 count from (**A**) pediatric KS (*n* = 21) versus (**B**) adult KS (*n* = 207). (**C**) KSHV viral load in pediatric KS compared with adult KS (*n* = 207). The dotted line represents the limit of detection at 1,000 copies/mL. (**D**) IL-6 and IL-10 levels on patient plasma with pediatric KS, measured by ELISA. Red and blue represent elevated levels of IL-6 and IL-10, respectively (above 100 pg/mL). Each data point represents the mean of *n* = 3 independent measurements, and the median was calculated for the entire group (unpaired 2-tailed *t* test; **P < 0.01). (**E**) KSHV, (**F**) IL-6, and (**G**) IL-10 levels in plasma from pediatric patients with KS at different time points. Only patients with 3 or more visits were included. Yellow and blue represent patients with elevated levels of KSHV and IL-10. Green represents a patient with elevated levels of IL-6, and red represents a patient with moderate levels of KSHV, IL-6, and IL-10. Patients marked in gray had no significant levels of any marker. Each data point represents the mean of *n* = 3 independent measurements.

To test the hypothesis that pediatric KS presents with a different pathobiology or is most closely associated with a subset of adult KS, fundamental KSHV pathogenesis features, such as viral loads and cytokine profile, were compared between this pediatric KS cohort (*n* = 34) and adult patients with KS seen at the same location between 2008 and 2019 (*n* = 207). Like in the US (30, 31), approximately one-third of adult HIV^+^ KS cases in Malawi present with an undetectable HIV viral load. The pediatric group did not recapitulate this trend. All pediatric KS cases presented with substantial HIV viral loads, and HIV viral load and CD4 counts were not correlated (coefficient of determination r^2^ = 0.023, with *P* ≤ 0.08 by *F* test; Figure 2A, pediatric). By comparison, adult patients with KS presenting at the same location during the observation period showed the expected negative relationship between HIV viral load and CD4 count (coefficient of determination r^2^ = 0.083, with *P* ≤ 0.0002 by *F* test; Figure 2B, adult).

Pediatric patients with KS tend to have a larger absolute amount of circulating immune cells (including T cells). Hence, it is crucial to consider the total cell count of these patients. This measure was available for only a subset of participants. When the HIV viral load was compared with the percent of CD4 cells rather than total CD4 counts, a negative correlation was observed (Supplemental Figure 2).

Pediatric KS cases had significantly higher KSHV viral loads at presentation (*P* value = 0.003) with a difference between means of (pediatric versus adults) 14,505 ± 4,815 copies/mL (Figure 2C). The pediatric group had a mean of 19,172 genome copies/mL, while the adult groups for 2008–2010 and 2017–2019 had a mean of 5,129 and 7,958 KSHV copies/mL, respectively.

Previous studies have associated high IL-10 and IL-6 with pediatric KS (15, 27). These observations were confirmed in the current cohort: IL-6 was present at 16.04 ± 29.11 pg/mL (mean ± SD, median = 8.50 pg/mL, *n* = 34), IL-10 at 96.87 ± 170.7 pg/mL (mean ± SD, median = 6.55, *n* = 34) at baseline, respectively (Figure 2D). A single patient had highly elevated levels of IL-6 with 166.8 pg/mL marked in red, and 6 patients had elevated IL-10 with concentrations ranging from 207.2 to 772.0 pg/mL marked in blue. Patients with extremely high levels of IL-6 did not have elevated levels of IL-10 and vice versa.

Collecting blood from pediatric patients is difficult. Repeat draws can only be justified under limited circumstances. Hence, we measured KSHV genome copy numbers, IL-6, and IL-10 levels over time for only 7 participants with 3 or more visits (Figure 2, E–G). KSHV genome copy numbers showed a similar trend as levels of IL-10 (patients marked in yellow and blue). The patient marked in green had extremely high levels of IL-6, but no other marker was elevated. Patients marked in gray did not have any elevated markers. These markers did not correlate with clinical features.

### Transcription of KSHV in pediatric KS.

**Figure 3:**
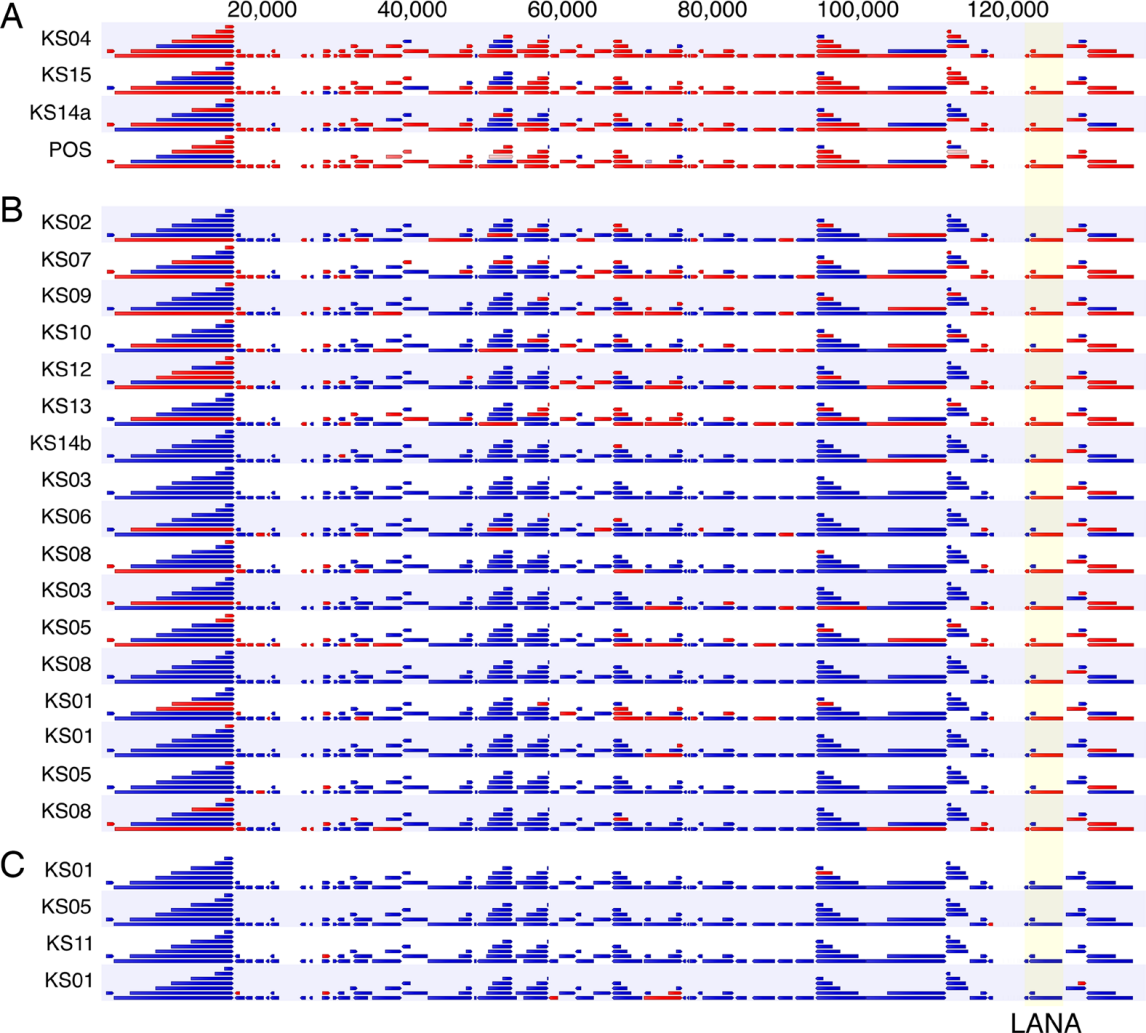
Viral RNA-Seq analysis of pediatric KS biopsies. RNA-Seq analysis of 25 pediatric KS biopsies; samples were grouped based on LANA expression. Red indicates transcripts are present, and blue indicates genes are absent. The numbers on top represent genome coordinates. Samples PEDKS01, PEDKS03, PEDKS05, and PEDKS08 had multiple libraries made and analyzed separately but were merged prior to performing human RNA-Seq analysis. PEDKS14a and PEDKS14b represent 2 different biopsies on the same participant. Positive (POS) is reactive BCBL-1 cell line RNA. Letters A–C indicate transcription clusters.

To determine KSHV transcription in pediatric KS, we performed RNA-Seq analysis. Figure 3 depicts the viral transcripts (32). Arrows colored red indicate active transcription across the locus, ≥3 reads per kilobase of transcript per million mapped reads (RPKM), and arrows colored blue indicate the absence of transcription. Samples were grouped based on LANA mRNA levels. All biopsies except 4 (cluster C in Figure 3) transcribed the KSHV latency locus that encodes the LANA protein (33), consistent with the histochemical detection of LANA. Note, however, that due to the small size of most biopsies, RNA-Seq and histological analysis were conducted on different lesions. The 4 biopsies that failed to yield a latency mRNA signal likely represent necrotic lesions or miss-sampling. Three lesions plus the positive control exhibited transcription across most annotated KSHV genes, a pattern characteristic of lytic viral replication; *n* = 17 had evidence of latent transcription but few detectable transcripts across most KSHV genes, most likely representing lesions in which KSHV persists latently. This distribution mirrors the pattern of viral transcription observed in adult KS (34).

**Figure 4:**
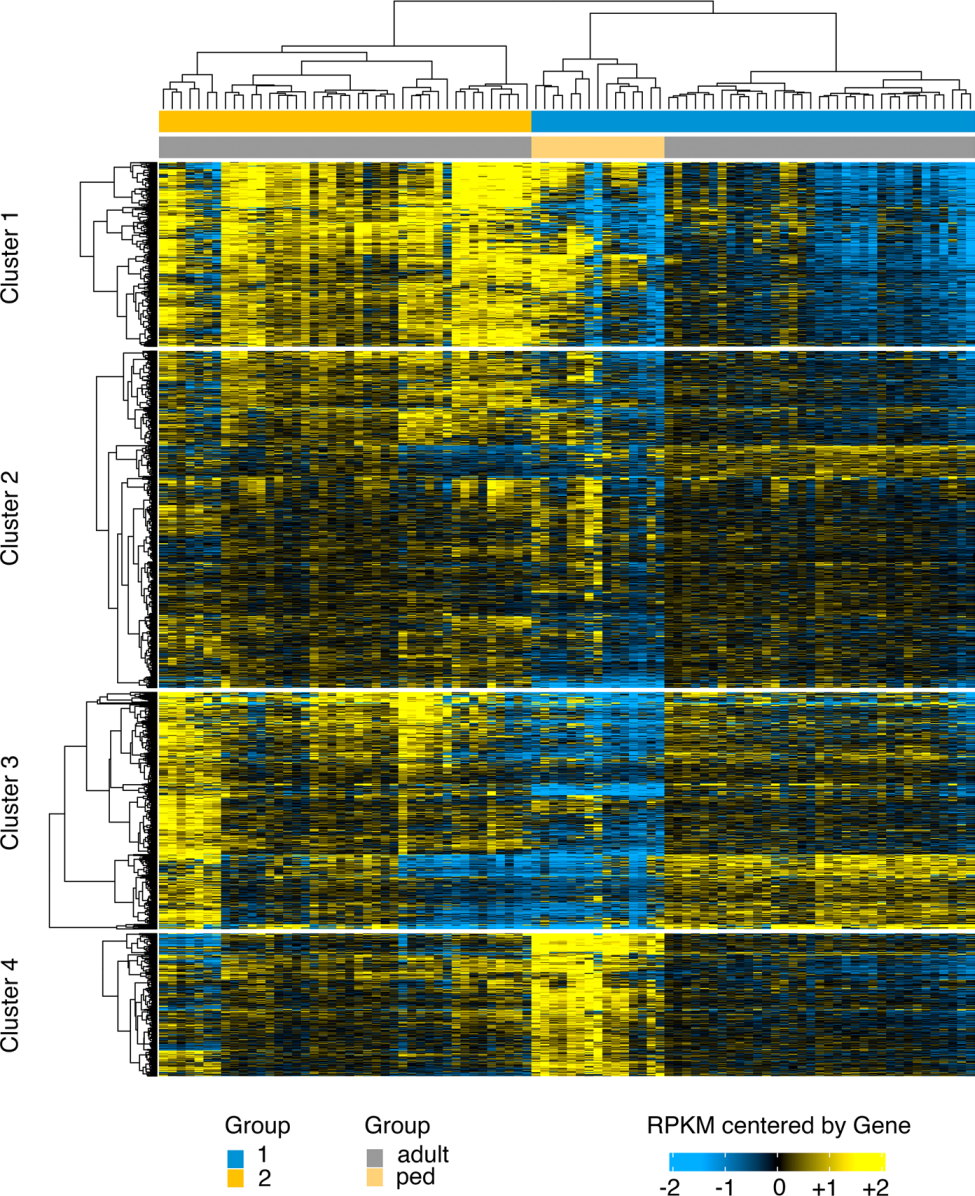
Human RNA-Seq heatmap of pediatric KS compared with adult KS. Shown is a heatmap of the log_2_ of RPKM data for protein-coding genes subjected to unsupervised hierarchical clustering using a Euclidian distance metric and Ward linkage. The scale refers to RPKM data that have been median centered by gene for the top 2,000 variable genes. Adult KS samples were separated into 2 subgroups: 1 in blue and 2 in yellow. All genes were clustered into 4 groups based on similarity scores.

To study the role of the host in the differential clinical representation of pediatric KS, we compared the human mRNA levels of pediatric KS biopsies (*n* = 15) to adult KS biopsies (*n* = 77) (Figure 4). Samples that had no detectable KSHV transcripts were removed before analysis. Adult HIV^+^ KS lesions clustered in 2 subtypes: group 1 (blue) and group 2 (yellow). These are described in Supplemental Table 5 and elsewhere (35). The pediatric KS samples formed a tight grouping within adult subgroup 1. This grouping is easily seen by Principal Components Analysis (PCA) (Supplemental Figure 4). PC2 nicely separates adult from pediatric cases. Note, however, that PC1 only explains 28.5% and PC2 only 17.8% of the variance. The majority of the variance is in the higher PCs, indicating a complex clustering. The differences between adult and pediatric KS were marked by 43 dramatically differentially expressed genes with a log_2_ fold change greater than 10 and an FDR-adjusted –log_10_ *P* value greater than 99 (Supplemental Figure 3A and Supplemental Table 4). The g:Profiler tool (36) was used to map the differently regulated genes to known pathways. The gene list was dominated by mitochondrial genes. The most enriched pathway was aerobic mitochondrial respiration (Supplemental Figure 3B). This was concerning, as an overrepresentation of mitochondrial genes may indicate dying cells in the sample. Since the samples were collected directly into RNAlater, this could not have been a technical issue, but it may be that pediatric KS lesions have higher rates of cell death or higher rates of metabolic activity. Therefore, a gene list without mitochondrial genes was queried. Translation emerged as the most dominant pathway (Supplemental Figure 3C) due to a preponderance of ribosomal proteins and the inclusion of EEF1G and EIF3. When the analysis was rerun without ribosomal genes, no significant pathway emerged. Many enriched proteins, such as IFITM1, CD59, and NOTCH2NL, are localized to the cell membrane. Together, these gene expression patterns paint the picture of highly metabolically active lesions proliferating and dying at an increased rate as compared with adult KS. Whether that signature stems from the tumor cell itself or reflects an influx of inflammatory cells cannot be determined by bulk RNA-Seq. The distinct gene expression between pediatric and adult KS highlights the differences between these diseases.

### KSHV genomes from pediatric KS.

We also determined the complete genomic sequence of KSHV from 11 biopsies (Supplemental Table 3). The read median was 9,715,313 ± 4,526,909 reads, and the median genome coverage was 90.94% ± 8.06% at 10x and 84.72% ± 9.91% at 50x. Six regions were not covered by this AmpliSeq array. A total of 162x ± 13x (median ± SD) viral Single Nucleotide Variants (SNVs) per genome were identified; however, no insertions and deletions (InDels) or SNVs were common to all genomes. This assured us that there was no contamination across samples. This is consistent with other studies documenting similar population variance of KSHV in KS endemic regions (37–39).

**Figure 5:**
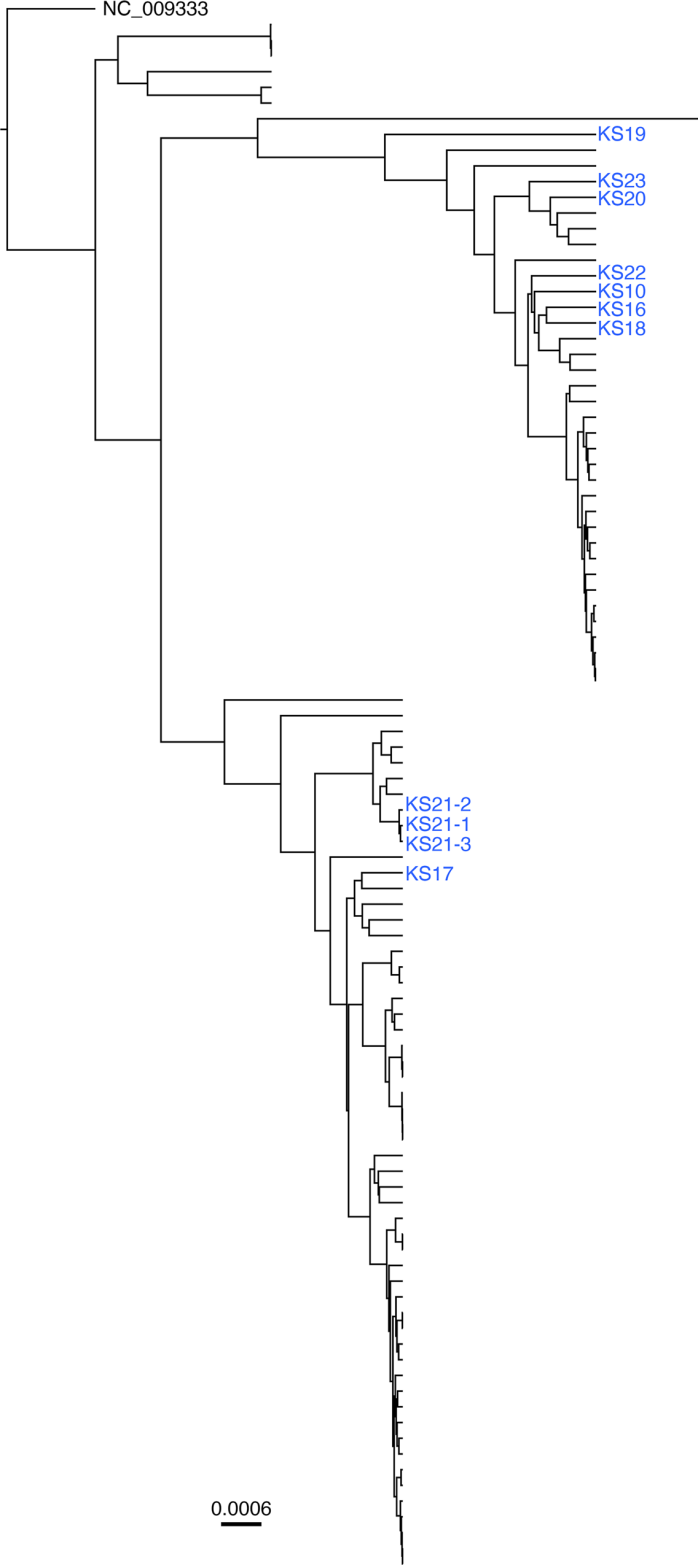
Phylogenetic analysis for pediatric KS genomes. Phylogenetic trees demonstrating alignment of the long unique regions of consensus sequences for KSHV isolated from pediatric KS biopsies compared with previously published KSHV consensus. Pediatric KS sequences are marked in blue. The ML tree contains *n* = 100 sequences and was rooted on NC_009333.

The 65,846 bp long unique region (LUR) region of 100 full-length KSHV genomes presently available in GenBank was used to construct a phylogenetic tree using Beast (40). The 11 pediatric cases aligned with what we previously termed clades A and B (Figure 5). These predominate in SSA and are distinct from isolates collected in the US and Western Europe during the 1990s (37). For 1 participant (PEDKS21), 3 different biopsies, PEDKS21-1, PEDKS21-2, and PEDKS21-3, yielded high-confidence genomes. These aligned more closely to each other than any other KSHV genome. This result validates our sequencing technique and consensus calling. It suggests that for this patient, a single mother-to-child infection event took place and that intrahost evolution, superinfection, or recombination was limited.

## Discussion

KS is about as common in SSA as prostate cancer is in the US. This is due to the high prevalence and early acquisition of KSHV and the unresolved HIV epidemic on the African continent (reviewed in ref. 41). Because in endemic regions, KSHV is acquired in early childhood, and because there are still instances of mother-to-child transmission for HIV, pediatric KS remains a common childhood cancer (13). Sufficiently validated treatment guidelines for pediatric KS do not exist, and our knowledge about the pathobiology of the disease is rudimentary. Yet, it is almost universally accepted that the biology of childhood cancers and early life immunity does not resemble that of adults (42, 43). Hence, there exists an urgent need to understand the molecular pathology of this disease.

Our experience treating and investigating pediatric KS cases in Malawi supports this contention (15, 27). The clinical presentation of pediatric HIV^+^ KS cases differs from that of adult HIV^+^ KS at the same location, and adult KS encountered in KSHV endemic regions today differs from the end-stage AIDS-KS described in the US in 1989 (44). The most striking difference between adult and pediatric KS is lymph node involvement. In this cohort, 71.4% of participants presented with bulky lymphadenopathy, most without discernible skin lesions.

Earlier, smaller studies investigated clinical and epidemiological parameters of pediatric KS (15, 19, 27, 45–48). We expand upon this work by characterizing viral and molecular characteristics of pediatric KS in the largest pediatric KS cohort to date. This study affirms LANA histochemistry as the defining diagnostic criterion for KS (49, 50).

Adding to earlier work by us and others (24, 37–39), we assembled full-length KSHV genomes with high coverage. These are the only KSHV genomes from pediatric KS. This information contributes to a long-term effort by the KSHV community to describe natural variations in this virus, which is essential to understanding the biology of the viruses circulating today and may inform KSHV vaccine efforts (51). We postulate the existence of at least 2 KSHV clades currently cocirculating in SSA. These 2 clades differ in the nucleotide level from earlier European isolates and primary effusion lymphoma isolates. However, it is unclear whether and how these coding differences (~ 140 nonsynonymous SNVs to the 1999 reference genome, assembled from a classical KS lesion) translate into different biological and clinical behaviors of KSHV.

Viral transcription in pediatric KS followed a similar pattern to adult HIV^+^ KS (32, 34, 52, 53). There exist 2 types of KS lesions: first, those with tightly restricted latent transcription, including LANA, K15, the viral IFN regulatory factors, and all viral miRNAs; and second, those with extended viral transcription, including RTA/ORF50, the viral IL-6 protein, and various early and late genes (52). In between, there are graduations, which we attribute to technical limitations concerning the sensitivity of the assays or abortive lytic replication. These 2 classes of KS lesions were previously described by *in situ* hybridization (33, 54). The biological relevance of these 2 types of KS lesions is unclear. Still, this molecular insight may explain the clinical observation that not all KS lesions respond similarly and with similar timing to therapies.

Interestingly, the 3 patients expressing a lytic KSHV pattern (Figure 3) also had lymphadenopathic (LN) KS and suffered from severe anemia (2.8–5.4 g/dL) and thrombocytopenia (platelet counts of 6–39). At this point, the data are sparse. Still, one could speculate that viral lytic pattern and LN KS association suggest that the disease pathology may be driven by the lytic phase of KSHV, which partially explains the clinical overlap between patients with LN KS and those with KICS.

For the first time, we could profile human transcription in pediatric KS and compare it to transcription patterns for adult KS. The transcription pattern for the pediatric KS lesions was similar but distinct from adult KS. Unfortunately, not enough lesions gave enough high-quality RNA to derive a pediatric KS signature or to identify individual biomarkers.

The circulating KSHV viral loads in pediatric patients with KS were higher and more consistent than in adult KS. At this point, we can only speculate about the physiological significance of this finding. Potentially, the adaptive immune response in children was less effective due to fewer lifetime exposure events, allowing for greater systemic viral replication. Perhaps KSHV-specific innate immune responses were less developed in early childhood or there was greater immune exhaustion due to competing infections and incomplete HIV suppression. We do not know if restricting KSHV replication would have clinical benefits in pediatric HIV^+^ KS. Nevertheless, the consistently high KSHV viral load provides an opportunity for molecular diagnosis in patients where biopsies are problematic.

IL-6 and IL-10 were often but not consistently elevated in pediatric KS. These 2 cytokines are deeply involved in KSHV pathobiology. They are part of the diagnostic criteria for multicentric Castleman disease (MCD) and KICS, which are seen concurrently with HIV^+^ KS (18, 24, 55). For idiopathic KSHV^–^ MCD, anti–IL-6 biologics (tocilizumab) are considered the first-line therapy (56), although tocilizumab had limited activity in KS (57). Following these 2 markers in pediatric KS may be helpful and feasible. Evidence is emerging that pomalidomide, which was recently approved for treating adult KS (58, 59), and lenalidomide suppress these and other cytokines in the context of HIV^+^ KS (60). Cytotoxic chemotherapy typically has a similar suppressive effect on these 2 cytokines. One can speculate that suppressing superphysiological cytokine levels in pediatric KS may have clinical benefits even if KS skin lesions do not visibly change.

This study has several limitations. First, there are numerous, well-recognized challenges in caring for patients in LMICs. The economic burden is severe, particularly on families with children, to participate in clinical trials. A lack of clinical infrastructure, timely access to treatment, and treatment resources present significant challenges to patient care. Additionally, the massive volume of patients relative to the number of clinicians and ancillary staff presents challenges to the comprehensive execution of research studies. Therefore, we could not reliably associate clinical outcomes with clinical interventions or molecular markers. Second, the technical difficulties of obtaining high-quality, robust, reproducible, and reliable molecular information on KS, particularly pediatric KS, are formidable. Biopsy specimens were obtained to achieve definitive histologic confirmation of the child’s malignancy. They were limited in size because of the feasibility and safety of the diagnostic procedure.

There are confounders that may introduce additional systematic or batch variation. All KS skin lesions, particularly on pediatric participants, are sampled to minimize bleeding and discomfort for the patients. This may result in a sample bias of skin versus lymphatic biopsies. Adult patients, because of economic constraints or less attention to overall health symptoms, wait possibly months before presenting to the tertiary hospital for a biopsy. By contrast, pediatric patients are closely monitored by their mothers, and pediatric KS has a different clinical course. Hence, pediatric patients may present with lesions at different stages of disease progression than adult patients.

Since KS lesions are heterogeneous regarding cellular and extracellular composition, this introduces additional biases, such as the fraction of viable tumor cells from which to derive a representative mRNA profile and the overall quality of the RNA. This study rejected samples with obvious quality issues and used an established algorithm to correct for technical variation; however, the transcriptional analysis also used data from other studies. On the one hand, such an experimental design increased the robustness as sample size increased; on the other hand, it may have introduced biases of unknown origin.

Repeat research biopsies, particularly in the pediatric population, are generally not allowed by the local institutional review board, as they offer no patient benefit. Because of the small size of the biopsy collected in routine clinical settings, we were not able to split the individual samples. Thus, this study did not match the transcriptional profile to histologic information and also did not generate replicates to validate genes individually by orthogonal methods. Clinical studies to address these limitations are ongoing.

Because of the nature of KS, it is unlikely that the chosen KS lesion reflects the status of the disease overall and, therefore, can become a predictive biomarker; however, the value of measuring molecular events in the tumor increases our understanding of the general pathobiology of the disease and the discovery of targets for intervention. Systemic viral load and cytokine levels in the blood seem more promising for developing a biomarker for pediatric KS. Measuring KSHV genome copy numbers, IL-6, and IL-10 may not produce a fully fledged classifier. Still, it may have utility in alerting the treating physician to impending complications or treatment failures, such as concomitant KICS or MCD. We must develop designs and devices that operate by finger-prick in LMIC environments. Further studies in pediatric KS are urgently needed, and it is encouraging to see efforts extended in this direction, such as probing the efficacy of paclitaxel, pomalidomide, liposomal doxorubicin, and propranolol in pediatric HIV KS.

## Methods

### Samples.

Seventy participants with pediatric KS were enrolled between June 2013 and August 2019 at Kamuzu Central Hospital in Lilongwe, Malawi. Biopsies were obtained in each case and evaluated by LANA staining to confirm the diagnosis. Additionally, blood samples were used for cytokines and viral load testing. Blood samples were collected in EDTA tubes, and plasma was isolated and frozen for storage. Samples were shipped in bulk on dry ice from Malawi to UNC Chapel Hill.

### HIV status.

HIV serostatus for patients 18 months and older was determined by rapid Ab test kits that were procured by the Malawi Ministry of Health. For those patients who are younger than 18 months, HIV qualitative PCR was used to confirm HIV status. HIV PCR was intended to be collected at baseline, at 6 months, and 12 months after enrollment. However, due to test reagent limitations, these were not always available, and therefore, many patients are missing data at some or all time points.

### LANA histochemistry.

Histochemistry was conducted for clinical purposes by the local pathology lab using commercially available reagents. Staining was repeated at UNC Chapel Hill using the Ab HHV8-LNA-L-CE (Leica) as per our prior publications (21).

### DNA extraction.

Tumor DNA was extracted using the QIAamp DNA FFPE Tissue Kit (Qiagen) per manufacturer protocol with 3 hours of proteinase K digestion (20 μL/sample) to increase DNA yield. PBMC DNA was extracted using a Roche MagNA Pure Compact Instrument (Roche, catalog 03731146001) and Nucleic Acid Isolation Kit I -Large Volume (Roche, catalog 03730972001). To serve as a control for DNA extraction efficiency, 10 μg of a control plasmid, Fly 2.0 (Addgene, catalog 117418), was added to each sample.

### ELISA.

IL-6, IL-8, and IL-10 were quantified as previously described (24) using the ready-set-go kit (eBioscience) per manufacturer instructions. Plates were washed with a plate washer ELx405 (Biotek). Absorbance was measured at 450 nm using a spectrophotometer Infinite M200 PRO (Tecan).

### Viral loads.

Following DNA extraction, real-time qPCR was performed to determine the genome copy number. Primers were obtained from Eurofins Genomics and used at 125 nM final concentration: KSHV, LANA78-F GAGCCCATAATCTTGCACGG and LANA78(2)-R GCCTCATACGAACTCCAGGT; EBV (61), EBNA3C-F AAGGTGCATTTACCCCACTG and EBNA3C-R AGCAGTAGCTTGGGAACACC; and Fly, Flyflap-f AATCATAAAGCGTTTTAAGCTCCAACGA and Fly Flyflap-r AATCATAATTCCTGACTCCCAAGTGGAC. Reactions were recorded with a Quant Studio 7 Pro (ABI) instrument. Cycle conditions were 95°C: 2 minutes (95°C, 15 seconds; 58°C, 1 minute; ×40).

### KSHV sequencing.

Total DNA was quantified by Qubit 3.0 dsDNA HS Assay (Life Technologies). Ion AmpliSeq primer pools (Life Technologies) were designed to amplify the viral genome based on KSHV BAC16 (JSC1 isolate, GenBank accession number GQ994935). Samples were sequenced on the Ion Torrent S5 (Life Technologies) with default parameters. Ion Torrent barcodes and adapters were removed. Next, reads were trimmed by low-quality bps (quality limit = 0.05), including 40 nucleotides at the 5’ terminal and 10 at the 3’ terminal. Reads shorter than 50 nucleotides were filtered out. Longer reads were mapped to the KSHV genome (GenBank accession number NC_009333) using CLC Genomics Workbench v20.0.3 (Qiagen) with default parameters (length fraction of 0.5 and similarity 0.8). Nonspecific reads were ignored. Duplicate mapped reads were collapsed. SNVs, multiple nucleotide variants (MNVs), and InDels were called using the Basic Variant Detection tool with default parameters. SNVs with a Phred score greater than 20, a minimum frequency greater than 60%, a minimum coverage greater than 40, and a minimum forward/reverse balance greater than 0.05 are reported. Consensus sequences were built on CLC Genomics Workbench (v20.0.3) based on quality score voting, with low-coverage regions being defined as regions with a minimum of 3 reads. Low-coverage regions were filled from the reference sequence (NC_009333) and annotated.

### RNA-Seq.

Biopsy Tissues were stored in RNAlater at –80°C. Tubes were thawed and approximately 30 mg tissue was lysed in 2 mL screw-top tubes containing 300 μL RLT (Qiagen; RNeasy Fibrous Tissue Mini Kit, catalog 74704), 0.05% DX Reagent (Qiagen, catalog 19088) and 3.0 mm (about 0.12 in.) stainless steel balls. Tissues were homogenized for 3 minutes in a TissueLyser (Qiagen, catalog 85300) for an additional 2 minutes, if necessary. Quantity and quality were accessed by Nanodrop (Thermo Fisher Scientific), Qubit 3 (Life Technologies), and 4200 TapeStation (Agilent Technologies). Ribosomal RNA was removed using RiboMinus Eukaryote System v2 and/or Low Input RiboMinus Eukaryote System v2 (Life Technologies, catalog A15026 and/or catalog 15027). Library preparation was performed using the Ion Total RNA-Seq Kit v2 protocol (Thermo Fisher Scientific, publication MAN0010654, revision C.0). Libraries were sequenced on the Ion Gene Studio S5 Prime System. Both adult and pediatric KS cases were sequenced simultaneously in the same facility; however, while the adult cases were analyzed using a human exome target approach (Thermo Fisher Scientific; Ion AmpliSeq Transcriptome Human Gene Expression Kit, catalog A26325), the pediatric cases were analyzed using complete RNA-Seq (Thermo Fisher Scientific; Ion Total RNA-Seq Kit v2, catalog 4479789) according to the manufacturer’s recommendations.

Analyses steps included quality control using bbduk version 37.25, mapping to reference genome (GRCh38, STAR aligner v2.5.3a, gencode v22 annotations), with read counting on genes (summarize Overlaps, mode: intersection-strict, single-end). Raw counts were converted to RPKM. Differential gene expression was calculated using DESeq2, working with a simple interaction term for the model: design = ~ drug + clone × number. PCA, distance heat maps, and other figures were generated based on RPKM in R using DESeq2 (which includes batch correction and normalization) and ggplot2. Genes were considered differentially expressed by the Wald test, with an adjusted (Benjamini-Hochberg) *P* value of less than 0.05.

### Phylogenetic analysis.

The phylogenetic tree was constructed using previously published KSHV genomes of African isolates Zambia (35), Uganda (34), and Malawi (33), as well as genomes from this study. One hundred genomes were aligned using MAFFT, implemented in Geneious v9.1.8 with default parameters (62). The multiple alignments based on the 65,840bp LUR region (ORF16–ORF58) of KSHV were used to infer the Bayesian Maximum Likelihood (ML) tree as implemented in Beast v1.10.4 (40) under the HKY substitution model. The tree was constructed with a strict clock and a constant size coalescent restriction. The ML tree was rooted on NC_009333.

### Statistics.

Results are reported as mean ± SD. All cellular experiments were repeated, resulting in 3 complete biological replicates. An unpaired 2-tailed *t* test with Welch correction (not assuming equal variance) was used to compare groups.

### Study approval.

This study was approved by the Malawi National Health Sciences Research Committee, protocol 17/03/1750, and the BCM IRB, protocol H-41274. For children above 5 years of age, we obtained assent from the child as well as consent from the parental guardian. Both the consent and assent forms were available in English and Chichewa, the national language of Malawi.

### Data availability.

The raw sequencing data were deposited in the NCBI’s Sequence Read Archive (SRA BioProject PRJNA975091). The adult KS data are available under Bioproject PRJNA947563. Viral genome sequences have been submitted to GenBank (accession ORO074929, OR074930, OR074931, OR074932, OR074933, OR074934, OR074935, OR074936, OR074937, OR074938, and OR074939). All computations are available in the bitbucket repository: https://bitbucket.org/dittmerlab/2021_naderpediadricks/src/master/ Values for all data points in graphs are reported in the Supporting Data Values file.

## Author contributions

CCV designed and performed experiments, analyzed data, and wrote the manuscript. AJ performed experiments. AP and RM performed the formal analysis of the study data. TT and CLK provided laboratory samples. AS, WK, JV, CLM, RM, ECPG, LRC, PSM, and PNK were part of the research and investigation process. CEA, MES, and NWO provided financial support for the project. DPD and NKEM designed experiments, analyzed data, provided financial support, and contributed to the final version of the manuscript. All authors approved the final manuscript.

## Supplementary Material

Supplemental data

ICMJE disclosure forms

Supporting data values

## Figures and Tables

**Table 1:**
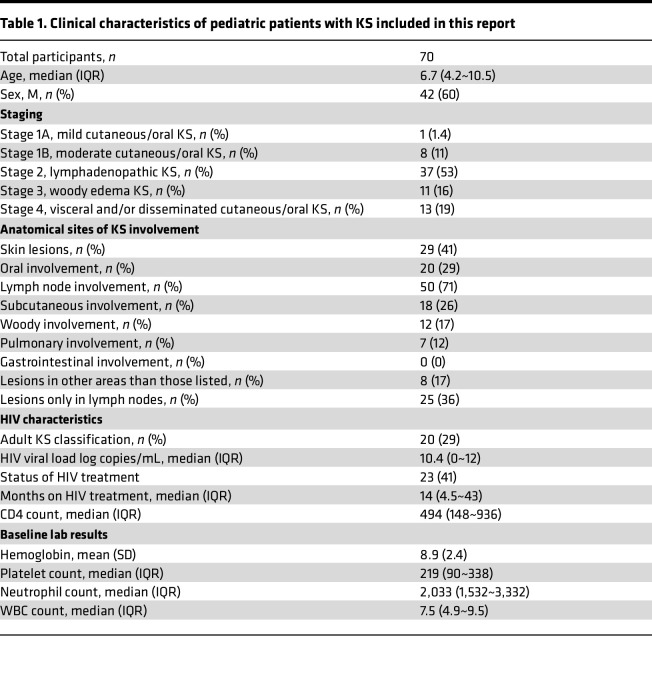
Clinical characteristics of pediatric patients with KS included in this report

## Version 1 11/22/2023

Electronic publication
