# Supplementary material for: Pediatric HIV+ Kaposi sarcoma exhibits clinical, virological, and molecular features different from the adult disease
Source: JCI Insight. 2023 Nov 22;8(22):e167854. doi: 10.1172/jci.insight.167854 (PMC10721314; doi:10.1172/jci.insight.167854)
Supplement: Supplemental data [file jciinsight-8-167854-s239.pdf]

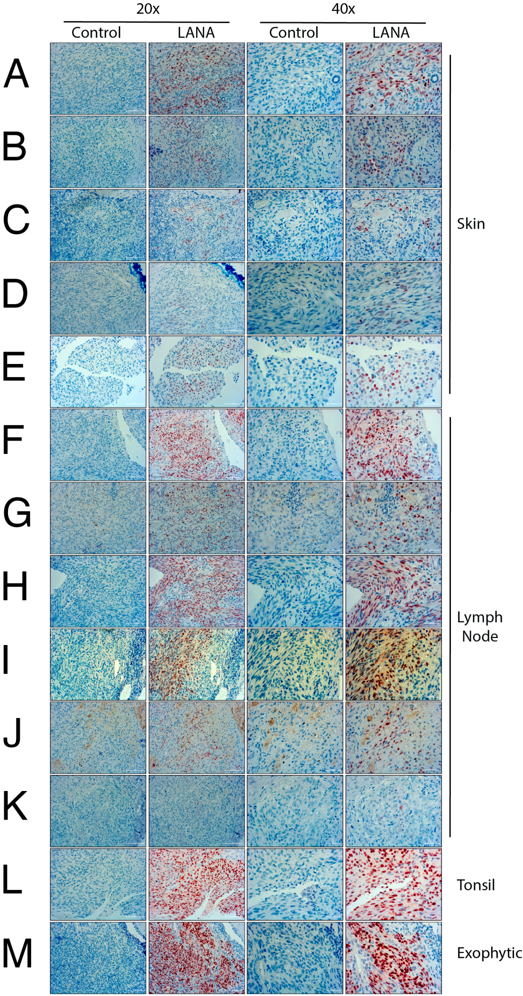

Sup. Figure 1: Representative images of pediatric KS biopsies staine with control or anti-LANA antibody at indicated figation. Location of the biopsy is on the right.

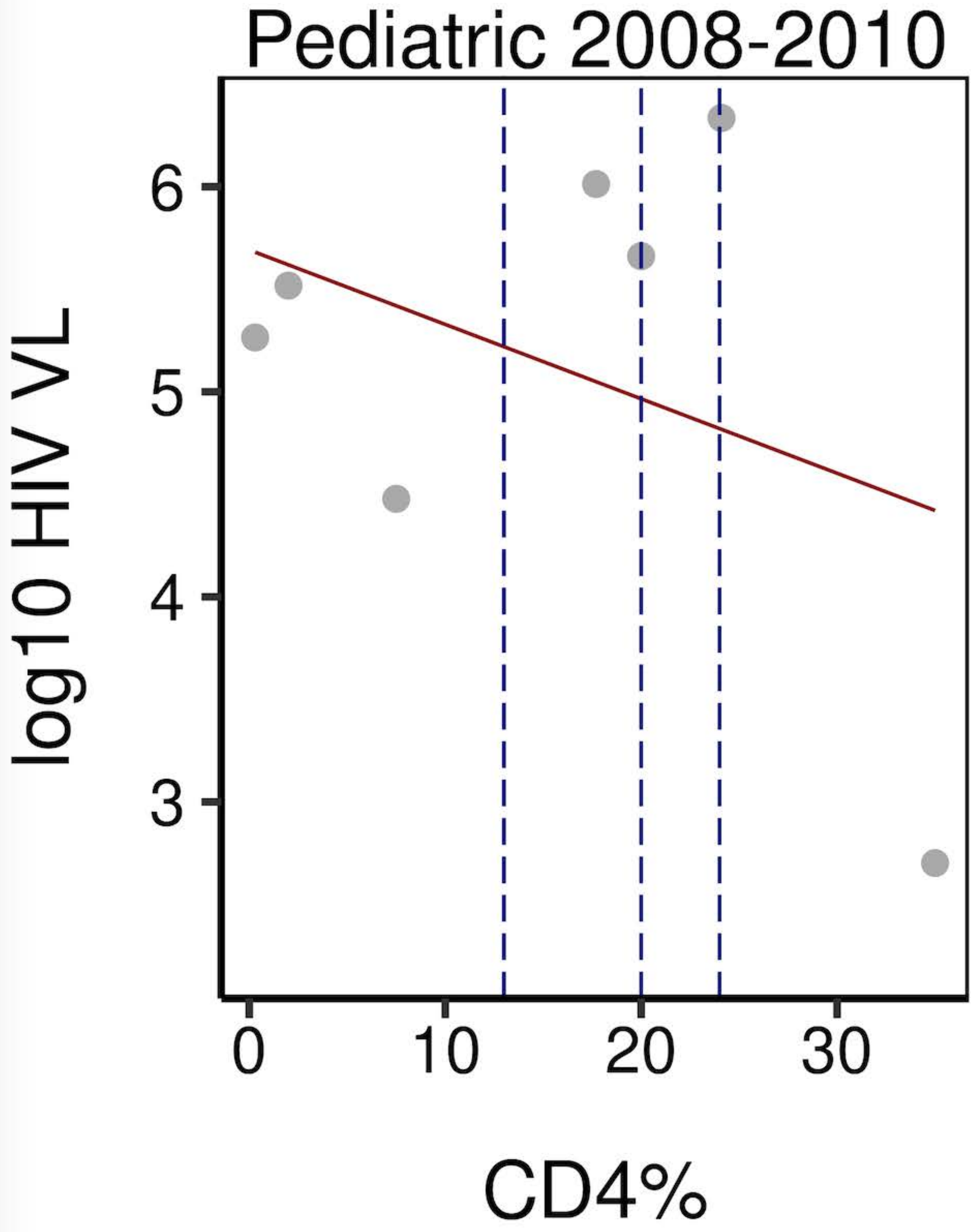

Sup. Figure 2: Shown are the log10 of HIV copy number on the vertical axis and the percent of CD4 cells of PBMC on the horizontal axis. Data are for pediatric participants, where available. The dashed lines indicate cutoff points. The red line indicates the linear regression.

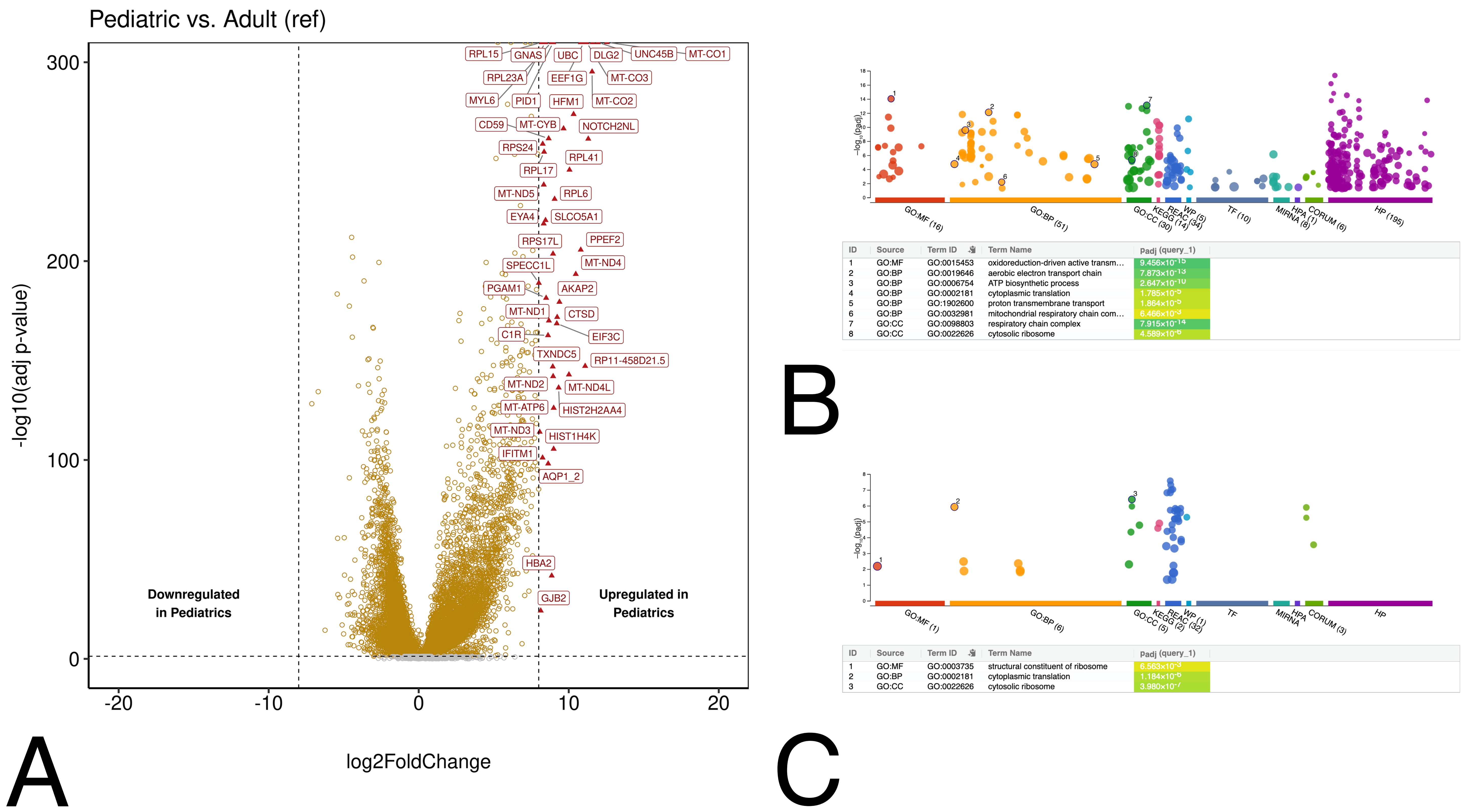

ID	Source	Term ID	Term Name	padj (query_1)
1	GO:MF	GO:0015453	oxidoreduction-driven active transm…	9.456×10^{-15}
2	GO:BP	GO:0019646	aerobic electron transport chain	7.873×10^{-13}
3	GO:BP	GO:0006754	ATP biosynthetic process	2.647×10^{-10}
4	GO:BP	GO:0002181	cytoplasmic translation	1.785×10^{-5}
5	GO:BP	GO:1902600	proton transmembrane transport	1.864×10^{-5}
6	GO:BP	GO:0032981	mitochondrial respiratory chain com…	6.466×10^{-3}
7	GO:CC	GO:0098803	respiratory chain complex	7.915×10^{-14}
8	GO:CC	GO:0022626	cytosolic ribosome	4.589×10^{-6}

ID	Source	Term ID	Term Name	padj (query_1)
1	GO:MF	GO:0003735	structural constituent of ribosome	6.563×10^{-3}
2	GO:BP	GO:0002181	cytoplasmic translation	1.184×10^{-6}
3	GO:CC	GO:0022626	cytosolic ribosome	3.980×10^{-7}

Supplemental Figure 3:
(A) Volcano plot displaying differential expressed genes between pediatric and adult KS. The y-axis corresponds to the mean expression value of log 10 (adj p-value), and the x-axis displays the log2 fold change value. The red triangles represent the most significant ($p < 1x10-6$) transcripts with differential expression between pediatric and adults KS. The grey dots represent the non-significant transcripts ($p > 1x10-6$) between pediatric and adults KS. Genes with log2fold change > 10 (1000-fold) are annotated. (B) Pathway analysis of the differentially regulated genes annotated in panel A. The most common GO-terms across multiple data bases are noted below the enrichment graph. (C) Pathway analysis of the differentially regulated genes excluding mitochondrial genes.

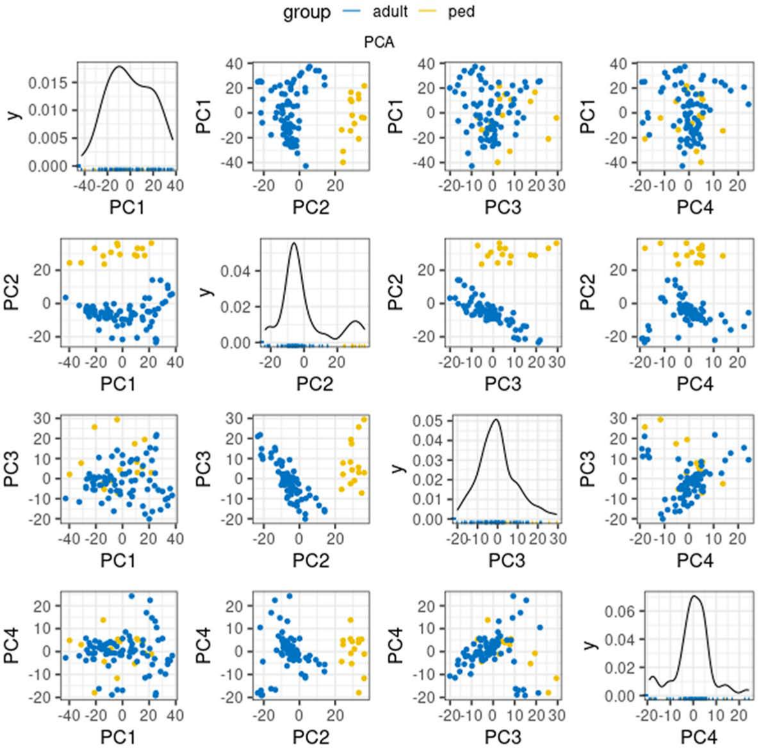

Supplemental Figure 4:
This is a PCA plot of the first four principal components directions showing that the adult KS. Samples cluster together and away from the pediatric samples.

Supplemental Table 1

Clinical characteristics of HIV negative participants

**Total Participants, n**	**14**
Age, median (IQR)	4.4 (3.4 ~ 9.5)
Gender, M, n (%)	9 (64.3)
**Clinical Characteristics**	
Mild Cutaneous/Oral KS, n (%)	0 (0)
Moderate Cutaneous/Oral KS, n (%)	3 (21.4)
Lymphadenopathic KS, n (%)	10 (71.4)
Woody Edema KS, n (%)	1 (7.1)
Visceral and/or Disseminated Cutaneous/Oral KS, n (%)	0 (0)
**Anatomical Sites of KS involvement**	
Skin lesions, n (%)	2 (14.3)
Oral involvement, n (%)	0 (0)
Lymph node involvement, n (%)	11 (78.6)
Subcutaneous involvement, n (%)	4 (28.6)
Woody involvement, n (%)	1 (7.1)
Pulmonary involvement, n (%)	0 (0)
Gastrointestinal involvement, n (%)	0 (0)
Lesions in other areas than ones listed, n (%)	4 (50)
Lesions only in lymph nodes, n (%)	9 (64.3)
**Baseline Lab Results**	
CD4 count, median (IQR)	2062(1575 ~ 2548)
Hemoglobin, mean (SD)	8.6 (2.9)
Platelet count, median (IQR)	215.5 (24 ~ 330)
Neutrophil count, median (IQR)	2543 (1882 ~ 4029)
White blood cell count, median (IQR)	7.5 (7.1 ~ 10.6)

Supplemental Table 2

Clinical characteristics of HIV positive participants.

**Total Participants, n**	**56**
Age, median (IQR)	7.1 (4.4 ~ 10.7)
Gender, M, n (%)	33 (58.9)
**Clinical Characteristics**	
Mild Cutaneous/Oral KS, n (%)	1 (1.8)
Moderate Cutaneous/Oral KS, n (%)	5 (8.9)
Lymphadenopathic KS, n (%)	27 (48.2)
Woody Edema KS, n (%)	10 (17.9)
Visceral and/or Disseminated Cutaneous/Oral KS, n (%)	13 (23.2)
**Anatomical Sites of KS involvement**	
Skin lesions, n (%)	27 (48.2)
Oral involvement, n (%)	20 (35.7)
Lymph node involvement, n (%)	39 (69.6)
Subcutaneous involvement, n (%)	14 (25)
Woody involvement, n (%)	11 (19.6)
Pulmonary involvement, n (%)	7 (15.6)
Gastrointestinal involvement, n (%)	0 (0)
Lesions in other areas than ones listed, n (%)	4 (10.5)
Lesions only in lymph nodes, n (%)	16 (28.6)
**HIV Characteristics**	
Adult KS classification, n (%)	19 (33.9)
HIV viral load log copies/mL, median (IQR)	4.7 (2.2 ~ 5.4)
Status of HIV treatment	23 (41.1)
Months on HIV treatment, median (IQR)	14 (4.5 ~ 43.5)
CD4 count, median (IQR)	398 (143 ~ 868)

Baseline Lab Results

**Total Participants, n**	**56**
Hemoglobin, mean (SD)	8.9 (2.3)
Platelet count, median (IQR)	219 (111 ~ 347)
Neutrophil count, median (IQR)	1,971 (1,334 ~3,258)
White blood cell count, median (IQR)	7.3 (4.5 ~ 9.4)

Supplemental Table 3

QC data for KSHV targeted sequencing analysis of KSHV genomes.

Samples	Total reads	Mapped Reads	10x Coverage	50x Coverage	SNVs#	Site	GenBank ID
PEDKS16	6,383,680	4,789,482	86%	75%	170	LN	
PEDKS17	17,555,865	16,291,744	94%	89%	129	LN	
PEDKS18	14,756,528	10,879,447	81%	71%	162	Cutaneous	
PEDKS19	12,963,395	9,602,310	68%	59%	158	Cutaneous	
PEDKS02	9,854,381	9,715,313	91%	86%	167	Exophytic	
PEDKS10	2,959,355	2,839,947	87%	81%	165	LN	
PEDKS21-1	14,995,909	14,876,729	95%	90%	150	Tonsil	
PEDKS21-2	16,269,326	16,212,125	95%	91%	160	Tonsil	
PEDKS21-3	9,273,912	9,218,664	94%	88%	155	Tonsil	
PEDKS22	5,688,369	5,585,763	92%	85%	180	LN	
PEDKS23	12,188,876	11,947,988	80%	77%	169	Edema	
Median	12,188,876	9,715,313	90%	5%	162		
St Dev	4,735,349	4,526,909	8%	10%	13		

(#) non-synonymous SNV

Supplemental Table 4

List of differentially regulated genes between adult and pediatric KS

Gene	Mean	log2 FoldChange	lfcSE	pvalue	padj	abs_log2 FoldChange
DLG2	26,106.20	11.60	0.29	0	0	11.60
EEF1G	491.94	11.01	0.28	0	0	11.01
GNAS	237.85	8.30	0.22	0	0	8.30
MT-CO1	3,122.12	12.54	0.24	0	0	12.54
MT-CO3	966.43	11.19	0.27	0	0	11.19
MYL6	222.82	8.85	0.23	0	0	8.85
PID1	4,528.65	8.96	0.23	0	0	8.96
RPL15	327.42	8.20	0.19	0	0	8.20
RPL23A	210.67	8.44	0.22	0	0	8.44
UBC	550.28	10.81	0.27	0	0	10.81
UNC45B	29,975.69	11.95	0.30	0	0	11.95
MT-CO2	997.86	11.56	0.32	5.44E-299	5.45E-296	11.56
HFM1	43,305.96	10.32	0.29	1.60E-277	1.35E-274	10.32
MT-CYB	439.19	9.65	0.28	2.82E-270	2.15E-267	9.65
CD59	210.98	8.66	0.25	3.10E-265	2.26E-262	8.66
NOTCH2NL	10,421.53	11.30	0.33	4.72E-265	3.29E-262	11.30
RPS24	230.70	8.27	0.24	1.82E-262	1.21E-259	8.27
RPL17	144.38	8.36	0.25	1.87E-258	1.20E-255	8.36
RPL41	386.25	10.05	0.30	2.30E-249	1.27E-246	10.05
MT-ND5	357.17	8.34	0.25	7.64E-242	4.08E-239	8.34
RPL6	277.95	9.06	0.28	1.23E-234	5.96E-232	9.06
SLCO5A1	8,632.37	8.45	0.27	6.17E-224	2.75E-221	8.45
EYA4	1,867.44	8.33	0.26	3.23E-222	1.40E-219	8.33
PPEF2	2,194.04	10.80	0.35	5.29E-209	2.12E-206	10.80
RPS17L	115.67	8.95	0.29	5.45E-207	2.03E-204	8.95
MT-ND4	768.93	10.47	0.35	1.09E-196	3.44E-194	10.47
SPECC1L	59.81	8.01	0.27	3.41E-192	1.01E-189	8.01
PGAM1	135.35	8.49	0.30	1.33E-184	3.44E-182	8.49
AKAP2	158.32	9.38	0.33	1.37E-182	3.44E-180	9.38
CTSD	193.62	9.23	0.33	6.52E-175	1.49E-172	9.23
MT-ND1	190.73	8.68	0.31	4.25E-173	9.21E-171	8.68
EIF3C	144.50	9.20	0.33	1.10E-171	2.30E-169	9.20
C1R	125.85	8.62	0.32	1.19E-165	2.19E-163	8.62
RP11-458D21.5	2,337.79	11.09	0.43	4.46E-150	6.06E-148	11.09
TXNDC5	120.98	8.94	0.35	8.84E-150	1.19E-147	8.94
MT-ND4L	273.82	10.01	0.39	9.12E-146	1.18E-143	10.01
MT-ND2	208.02	8.96	0.35	6.90E-145	8.71E-143	8.96
HIST2H2AA4	155.52	9.33	0.38	3.81E-139	4.40E-137	9.33
MT-ATP6	228.58	9.00	0.37	7.60E-129	7.04E-127	9.00
MT-ND3	72.22	8.06	0.35	1.06E-116	7.91E-115	8.06
HIST1H4K	125.49	8.99	0.41	4.61E-108	2.80E-106	8.99
IFITM1	79.75	8.26	0.39	1.36E-103	7.58E-102	8.26
AQP1_2	128.99	8.63	0.41	1.39E-100	7.18E-99	8.63

Supplemental Table 5

Clinical characteristics of adult KS samples used for RNAseq.

Number of participants (n)	78
Age, years, median (IQR)	36.9 (32 ~ 42)
Gender; men, n (%)	62 (79.5)
**Staging**	
Tumor severity T0 (ACTG), n (%)	12 (15.4)
On ART <3 months prior to KS treatment	5 (41.7)
Karnofsky performance status ≤70%, n (%)	31 (39.7)
Illness severity S1, n (%)	46 (59)
**Symptoms**	
Oedema present, n (%)	39 (50.6)
Visceral disease present, n (%)	6 (7.8)
Oral involvement present, n (%)	29 (37.7)
**HIV Characteristics**	
HIV diagnosis prior to KS diagnosis, n (%)	43 (55.1)
Months HIV+ diagnosis known, median (IQR)	15.7 (6.3 ~ 64.3)
On ART prior to KS diagnosis, n (%)	37 (47.4)
Months on ART prior to diagnosis, median (IQR)	15.7 (6.4 ~ 65.8)
CD4 count, median (IQR)	197 (103 ~ 337)
HIV viral load log copies/mL, median (IQR)	2.6 (1.6 ~ 4.8)
HIV viral load < 1000 copies/mL, n (%)	46 (61.3)
**Baseline Lab Results**	
Hemoglobin, g/dL, mean (SD)	11.3 (2.7)
Platelets, 103/3/μL, median (IQR)	230 (145 ~ 273)
White blood cell, x10/μL, median (IQR)	4.7 (3.8 ~ 5.8)
Absolute neutrophil count, $x10^3$/μL, median (IQR)	2.2 (1.5 ~ 2.8)
Creatinine, mg/dL/ median (IQR)	0.8 (0.7 ~ 0.9)
Bilirubin, total, mg/dL, median (IQR)	0.4 (0.3 ~ 0.5)
